# Die multiplanare v-förmige distalisierende Tuberositasosteotomie mit proximalem Widerlager („sargförmige“ Osteotomie)

**DOI:** 10.1007/s00132-021-04112-w

**Published:** 2021-05-04

**Authors:** F. Riechelmann, A. Wurm, D. Putzer, M. Ban, D. Dammerer, M. C. Liebensteiner

**Affiliations:** 1 Dept. für Orthopädie und Traumatologie, Medizinische Universität Innsbruck, Anichstraße 35, 6020 Innsbruck, Österreich; 2 Experimentelle Orthopädie, Medizinische Universität Innsbruck, Innsbruck, Österreich

**Keywords:** Kniegelenk, Patella, Kniescheibeninstabilität, Chirurgisches Verfahren, Tibia, Knee joint, Patella, Patellar dislocation, Surgical procedures, Tibia

## Abstract

Das Operationsziel des hier dargestellten Verfahrens ist eine Distalisierung der Tuberositas tibiae. Dieser Eingriff ist bei Patienten mit einer symptomatischen Patella alta indiziert, zum Beispiel bei Kniescheibeninstabilität. Die Osteotomie erfolgt v‑förmig. Der bei der Distalisierung gewonnene Knochenblock wird als proximales Widerlager eingebracht. Dadurch wird die mediolaterale und proximale Stabilität verbessert. Außerdem wird die Oberfläche vergrößert, was eine bessere Knochenheilung ermöglicht. Bei dem vom Operateur behandelten Patientenkollektiv von 10 Patienten ist es bei dieser Technik zu keiner sekundären Dislokation gekommen.

**Video online:**

Die Online-Version dieses Beitrags (10.1007/s00132-021-04112-w) enthält ein Video zur distalisierenden Tuberositasosteotomie mit proximalem Widerlager. Beitrag und Video stehen Ihnen im elektronischen Volltextarchiv auf SpringerMedizin.de unter http://www.springermedizin.de/der-orthopaede zur Verfügung. Sie finden das Video am Beitragsende als „Supplementary Information“.

## Abstract (en)

The aim of the intervention presented is a distalization of the tibial tuberosity. It is indicated in patients with symptomatic patella alta, i.e. patients with instability of the patella. It facilitates a V-shaped osteotomy. The bone gained during distalization is used as a proximal buttress. This leads to an improved mediolateral and proximal stability. The bony surface area is increased, which improves bony healing. There were no secondary dislocations in the patient group of 10 patients treated by the surgeon.

## Hintergrund und Operationsindikation

Die Ursachen der Kniescheibeninstabilität sind multifaktoriell. Neben einem insuffizienten medialen Bandapparat, der Trochleadysplasie, einem erhöhten „tibial tubercle-trochlear groove“(TT-TG)-Abstand und Achsrotationsfehlern spielt auch die Patella alta eine entscheidende Rolle [1]. Die Behandlung der Instabilität ist entsprechend komplex und erfolgt an unserer Klinik nach dem Algorithmus des AGA-Komitee-Knie-Patellofemoral [3]. Bei erhöhten TT-TG-Abständen ist der lateralisierende Zug der Patellasehne auf die Kniescheibe erhöht. Bei der Patella alta kommt es in den frühen Knieflexionsgraden zu einem verspäteten Kontakt der Kniescheibe mit der Trochlea femoris. Beides führt zu Instabilität [4]. Zusätzlich ist bei der Patella alta die patellofemorale Kontaktfläche reduziert, wodurch das Risiko für Arthrose steigt [8]. Sowohl die Patella alta als auch pathologische TT-TG-Abstände können an der Tuberositas tibiae (TT) korrigiert werden. Bei normwertigen TT-TG-Abständen kann eine isolierte Distalisierung der TT erfolgen.

Zur Bestimmung der Patellahöhe sind mehrere Messmethoden etabliert. Der Caton-Deschamps-Index (CDI) und der Blackburne-Peel-Index beschreiben den Abstand der Kniescheibe zum Tibiaplateau. Der Insall-Salvati-Index beschreibt den Abstand der Kniescheibenspitze zur Tuberositas tibiae, er verändert sich also bei Versatz der Tuberositas nicht. Ein Caton-Deschamps-Index von > 1,2 gilt als pathologisch [5]. Eine etablierte Behandlungsmethode ist die Distalisierung der TT. Beim operativen Standardverfahren erfolgt eine plane zweidimensionale Osteotomie, welche mit 2 Schrauben fixiert wird [2]. Hierbei kann die Tuberositas sowohl distalisiert als auch medialisiert werden. Aufgrund der fehlenden Abstützung nach proximal und seitlich ist die sekundäre Dislokation eine häufige Komplikation dieses Verfahrens [6, 7]. Zur Vermeidung dieser Komplikationen wurde ein operatives Verfahren entwickelt, welches eine multidimensionale v‑förmige Osteotomie mit proximalem knöchernem Widerlager nutzt. Die Vorteile des Verfahrens sind eine verbesserte knöcherne Abstützung nach proximal. Außerdem entstehen eine verbesserte Seitabstützung sowie vergrößerte knöcherne Kontaktflächen, um so das Einheilen des Knochens zu verbessern.

## Fallbeschreibung

Bei dem im Video dargestellten Fall handelt es sich um einen 17-jährigen männlichen Patienten mit habitueller patellofemoraler Instabilität und anamnestisch multiplen Patellaluxationen des linken Kniegelenks. Konservative Therapiemaßnahmen mittels Physiotherapie und Orthetik brachten keine Besserung. In der klinischen Untersuchung zeigt sich eine gerade Beinachse, „range of motion“ Extension/Flexion 0/0/120°, Kreuz- und Seitenbänder stabil, Instabilitätssymptomatik der Patella mit positivem Apprehension-Zeichen in den frühen Flexionsgraden von 0–30°, ab ca. 40°Knieflexion stabiler Patellalauf, positives J‑Zeichen, in Bauchlage kein Hinweis für femorale Maltorsion, keine „squinting patellae“, kein „in-toeing“. In der präoperativen Röntgenuntersuchung zeigt sich ein Caton-Deschamps-Index von 1,4. In der MRT zeigt sich eine Trochleadysplasie Typ A nach Dejour. Der TT-TG beträgt 13 mm. Eine bildgebende Rotationsanalyse wurde aufgrund fehlender klinischer Hinweise nicht durchgeführt.

## Präoperative Planung

**Figure 1:**
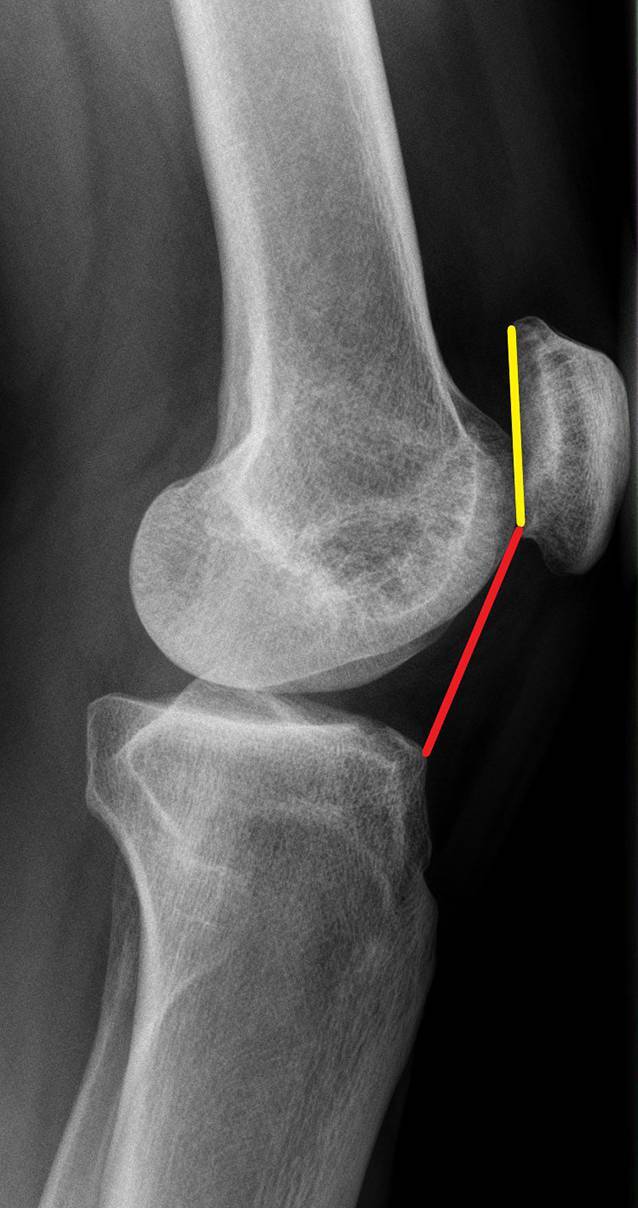

Nativradiologische Bestimmung des Caton-Deschamps-Index, virtuelle Distalisierung der Tuberositas tibiae mit einem geplanten Ziel CDI von 1,0 am betroffenen Kniegelenk. Die entsprechende Distalisierungsstrecke muss am distalen Osteotomieende subtrahiert werden. Der resezierte Knochen wird später proximal als Widerlager eingebracht. Eine Ratio von mindestens 3:1 sollte eingehalten werden. Sollte, zum Beispiel, eine Distalisierung von 1 cm notwendig sein, sollte die gesamte Osteotomiestrecke mindestens 4 cm betragen. Es entsteht entsprechend ein 1 cm langer Keil und eine 3 cm lange Tuberositas tibiae (Abb. 1 und 2).

**Figure 2:**
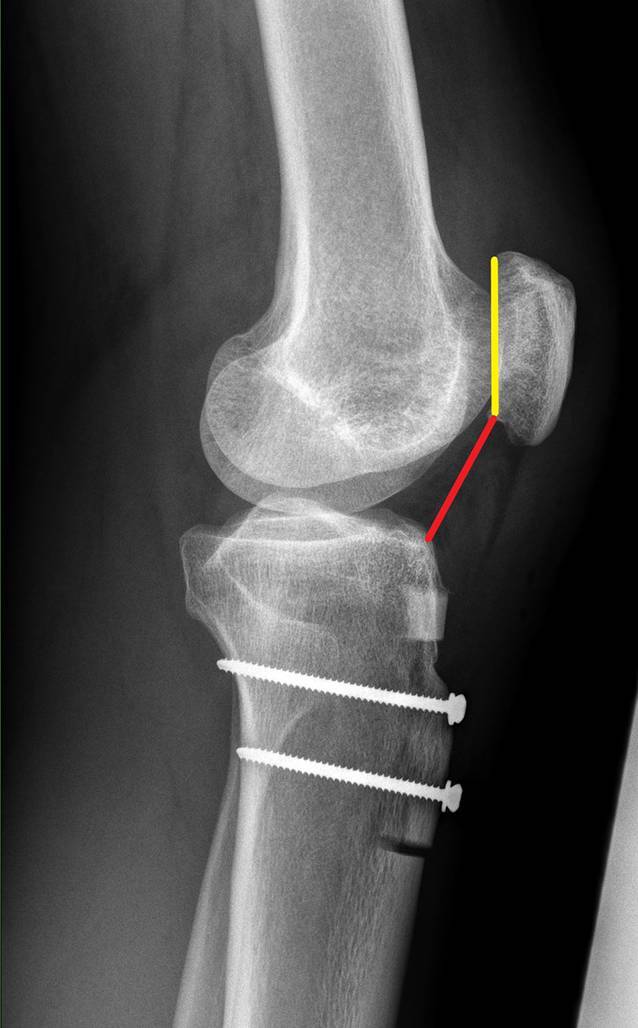

## Operationstechnik

**Figure 3:**
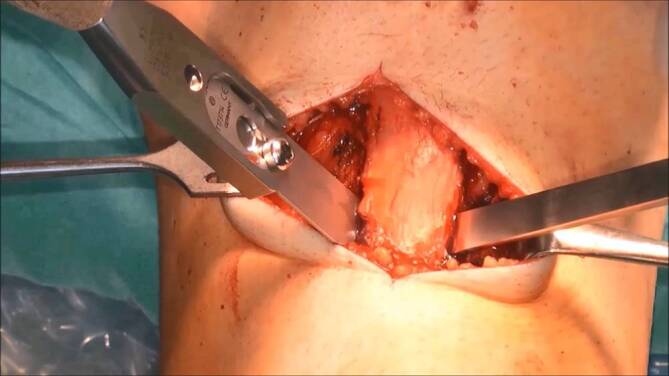

**Figure 4:**
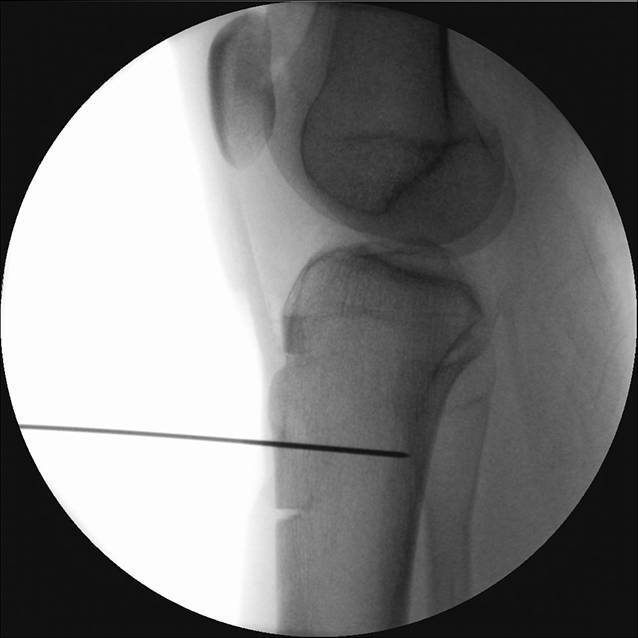

Die Patientenlagerung erfolgt mit Keilpolster und Seitstütze. Die Operation erfolgt in Blutleere. Die Haut-Weichteil-Inzision erfolgt longitudinal, beginnend an der Gelenklinie bis ca. 2 Querfinger distal des Tuberositasendes. Scharfe Dissektion bis auf die epifasziale Schichte. Darstellen des medialen und lateralen Rands des Ligamentum patellae (Video [m:s] 00:27). Unterfahren des Ligamentum patellae zur exakten Bestimmung des distalen Sehnenansatzes. Kurzstreckige Fasziotomie des anterioren Unterschenkelkompartments (Video 01:00). Geringfügiges Mobilisieren des Musculus tibialis anterior zur Darstellung der lateralen Fläche der Tuberositas tibiae. Fasziotomie der Fascia cruris entlang des medialen Randes der Tuberositas tibiae und Inzision des Periosts, vorbereitend für die Osteotomie. Abmessen der geplanten Osteotomiestrecke und Planung der Osteotomie im Sinne eines v‑förmig zu exzidierenden Keils (Abb. 3). Beginn der Osteotomie von medial, auf die laterodorsale Tibiakante zielend und Querosteotomie des distalen Endes. Vervollständigen der v‑förmigen Osteotomie von lateral mit Ziel auf die dorsale Tibiafläche (Video 01:20). Vervollständigen der Osteotomie mit dem Meißel, proximal unter Schonung des Ligamentum patellae, bis sich ein gleichschenklig trapezförmiges (sargförmiges) Segment heben lässt (Video 02:40). Mobilisation des Ligamentum patellae unter Schonung des Hoffa-Fettkörpers. Markieren der geplanten Distalisierungsdistanz und Osteotomie des distalen Knochenblocks. Einbringen des distal resezierten Knochenblocks in die proximale Defektstrecke. Behutsames Einbolzen des Knochenblocks mit dem Stößel. Die distalisierte Tuberositas wird mit einem Kirschner-Draht temporär in der gewünschten Position fixiert. Eine Bildwandlerkontrolle im seitlichen Strahlengang bestätigt die korrekte Position der Tuberositas und des Widerlagers (Abb. 4). Bikortikale Fixation des Segmentes mittels Kleinfragmentschrauben in Zugschraubentechnik (Video 04:33). Die korrekte bikortikale Schraubenlage wird abschließend unter Bildwandlerkontrolle im seitlichen Strahlengang dokumentiert (Video 05:35).

## Klinische Erfahrung

Das Verfahren wurde von einem einzelnen Operateur seit Anfang 2019 in der dargestellten Art an insgesamt 10 Patienten in einem Alter von 16–31 Jahren angewandt. Davon waren 6 männlich. Der Nachbeobachtungszeitraum betrug im Median 10,5 Monate. Im Beobachtungszeitraum kam es bei keinem der Patienten zu Osteosyntheseversagen oder sekundärer Dislokation.

## Fazit für die Praxis

- Die Behandlung von Patienten mit patellofemoraler Instabilität ist komplex.
- Neben Eingriffen an der Trochlea und Rekonstruktionen des medialen Kapsel-Band-Apparates ist der Tuberositastransfer ein etabliertes Verfahren. Aufgrund des starken Zugs der Patellasehne ist er jedoch mit einem hohen Risiko der sekundären Dislokation verbunden.
- Die beschriebene Operationstechnik zeigt eine alternative Möglichkeit, die Tuberositas tibiae zu distalisieren.
- Der klinische Verlauf von 10 Fällen ist bislang vielversprechend.
- Durch die vergrößerte Knochenkontaktflächen wird das Pseudarthroserisiko verringert, durch die Abstützung zur Seite und insbesondere nach proximal die Stabilität verbessert.
- Die Nachbehandlung erfolgt analog zum chirurgischen Standardverfahren.

## Supplementary Information



## Abkürzungen

AGA: Gesellschaft für Arthroskopie und Gelenkchirurgie
CDI: Caton-Deschamps-Index
TT: Tuberositas tibiae
TT-TG: „Tibial tubercle-trochlear groove“
